# Upconversion nanoparticle-mediated photodynamic therapy induces autophagy and cholesterol efflux of macrophage-derived foam cells via ROS generation

**DOI:** 10.1038/cddis.2017.242

**Published:** 2017-06-08

**Authors:** Xiaobo B Han, Hongxia X Li, Yueqing Q Jiang, Hao Wang, Xuesong S Li, Jiayuan Y Kou, Yinghong H Zheng, Zhongni N Liu, Hong Li, Jing Li, Dou Dou, You Wang, Ye Tian, Liming M Yang

**Affiliations:** 1 Department of Pathophysiology, Key Laboratory of Cardiovascular Pathophysiology, Harbin Medical University, Harbin, China; 2 Department of Food Science and Engineering, College of Food Science, Northeast Agricultural University, Harbin, China; 3 Department of Electron Microscopic Center, Harbin Medical University, Harbin, China; 4 Department of Physiology and Pathophysiology, School of Basic Medical Sciences, Peking University Health Science Center, Beijing, China; 5 Materials Physics and Chemistry Department, Harbin Institute of Technology, Harbin, China; 6 Division of Cardiology, The First Affiliated Hospital, Harbin Medical University, Harbin, China

## Abstract

Macrophage-derived foam cells are a major component of atherosclerotic plaques and have an important role in the progression of atherosclerotic plaques, thus posing a great threat to human health. Photodynamic therapy (PDT) has emerged as a therapeutic strategy for atherosclerosis. Here, we investigated the effect of PDT mediated by upconversion fluorescent nanoparticles encapsulating chlorin e6 (UCNPs-Ce6) on the cholesterol efflux of THP-1 macrophage-derived foam cells and explored the possible mechanism of this effect. First, we found that PDT notably enhanced the cholesterol efflux and the induction of autophagy in both THP-1 and peritoneal macrophage-derived foam cells. The autophagy inhibitor 3-methyladenine and an ATG5 siRNA significantly attenuated PDT-induced autophagy, which subsequently suppressed the ABCA1-mediated cholesterol efflux. Furthermore, the reactive oxygen species (ROS) produced by PDT were responsible for the induction of autophagy, which could be blocked by the ROS inhibitor N-acetyl cysteine (NAC). NAC also reversed the PDT-induced suppression of p-mTOR and p-Akt. Therefore, our findings demonstrate that PDT promotes cholesterol efflux by inducing autophagy, and the autophagy was mediated in part through the ROS/PI3K/Akt/mTOR signaling pathway in THP-1 and peritoneal macrophage-derived foam cells.

Atherosclerosis is a chronic inflammatory cardiovascular disease that is the leading cause of death in industrialized societies and worldwide.^1^ The internalization of modified low-density lipoprotein (LDL) by macrophages is the major process leading to foam cell formation and contributes to the inflammatory milieu and atherosclerotic plaque progression.^2, 3^ The alleviation of lipid deposition in macrophage-derived foam cells is a promising atherosclerosis treatment strategy. However, because pharmacological therapy has numerous limitations,^4^ the development of new treatments to attenuate lipid deposition is desirable.

Photodynamic therapy (PDT) is a therapeutic strategy for various diseases and involves three key components: a photosensitizer, light, and molecular oxygen.^5, 6, 7^ Although PDT has been applied clinically to treat diseases, its notable drawback is that the effect of the traditional photosensitizer chlorin e6 (Ce6) has a shallow depth.^8, 9^ In this study, we combined the widely used photosensitizer Ce6 with silica nanoparticles, which have high hydrophilicity, good biocompatibility and favorable optical properties, to form a UCNPs-Ce6 complex. Then, we utilized a 980-nm laser to enhance the penetration depth of the light and upconversion nanoparticles (UCNPs) to convert the 980-nm laser to short-wavelength visible emission light capable of directly activating the photosensitizers.^10, 11, 12^

As major products of PDT, reactive oxygen species (ROS) generation was able to induce mitochondrial dysfunction, leading to cell death.^13, 14^ Additionally, emerging evidence has confirmed that ROS are early inducers of autophagy.^15^ Autophagy is an evolutionarily conserved process that responds to cellular stress conditions to maintain a healthy cellular status by degrading and recycling cytoplasmic contents via the lysosomal route.^16, 17^ Numerous reports have shown that autophagy participates in the regulation of lipid metabolism and cholesterol homeostasis, with a special emphasis on macrophage-derived foam cells.^18, 19^

Based on the vital roles of autophagy in cholesterol homeostasis, we explored the effect of UCNPs-Ce6-mediated PDT on cholesterol efflux by activating the autophagic process via ROS generation. Therefore, the aim of this study was to investigate whether UCNPs-Ce6-mediated PDT contributed to cholesterol homeostasis via the activation of autophagy.

## Results

### Cell viability after various treatments

To choose the optimal PDT conditions, cell viability was determined after different treatments using the CCK-8 assay. A dosage of 8 *μ*g/ml of UCNPs-Ce6 was safe and had no significant effect on cell viability with increasing incubation time, whereas the survival rate decreased significantly at the 12 *μ*g/ml UCNPs-Ce6 concentration (Supplementary Figures 1a and b). Furthermore, cell viability decreased significantly at a laser density of 1.2 W/cm^2^ using a laser irradiation time of 60 s (Supplementary Figure 1c). Moreover, when the cells were subjected to a laser density of 1.0 W/cm^2^ with different laser irradiation times, the cell viability decreased significantly at a laser irradiation time of 80 s (Supplementary Figure 1d). Accordingly, 8 *μ*g/ml of UCNPs-Ce6 and 60 s of laser irradiation with a 1.0 W/cm^2^ capacity was selected as the optimal condition in which PDT had no significant effect on cell viability (Supplementary Figure 1e) and the optimal condition for peritoneal macrophage foam cells (Supplementary Figure 2).

### UCNPs-Ce6-mediated PDT promoted the cholesterol efflux of THP-1 macrophage-derived foam cells

**Figure 1:**
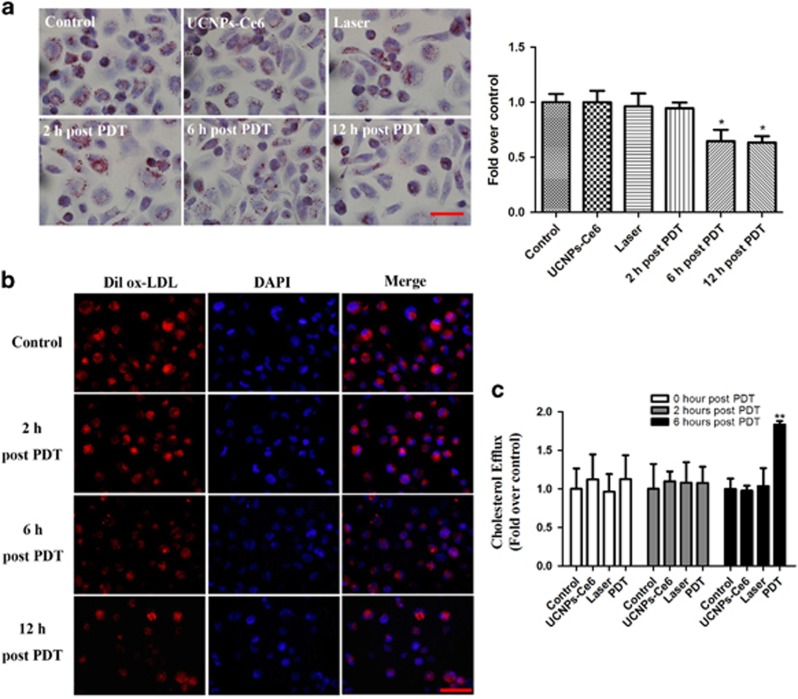
UCNPs-Ce6-mediated PDT promotes the cholesterol efflux of THP-1 macrophage-derived foam cells. (**a**) The effect of PDT on the intracellular lipid burden measured by oil red O staining (scale bar, 50 *μ*m). (**b**) The effect of PDT on the intracellular lipid burden measured by Dil ox-LDL and DAPI staining (scale bar, 50 *μ*m). (**c**) The quantitative detection of the cholesterol efflux following PDT using the fluorometric assay. (*n*=3; *P<0.05 *versus* control group, **P<0.01 *versus* control group)

To investigate whether PDT triggered cholesterol efflux, we examined lipid deposition and measured the cholesterol efflux. Oil red O staining indicated that lipid storage in cells decreased significantly starting 6 h after PDT (Figure 1a). We utilized Dil-labeled oxidized (ox)-LDL to trace the ox-LDL uptake and found that red fluorescence was attenuated starting 6 h post-PDT (Figure 1b). Furthermore, cholesterol efflux detected using a fluorometric assay also showed that the ratio of the fluorescence intensity in the medium to the total fluorescence intensity in both the cell lysate and the medium was higher in the PDT group than in the control, laser and UCNPs-Ce6 alone groups at 6 h post-PDT (Figure 1c). These results suggest that PDT promotes the cholesterol efflux of THP-1 macrophage foam cells.

### Cholesterol efflux of THP-1 macrophage-derived foam cells was correlated with autophagy induced by UCNPs-Ce6-mediated PDT

**Figure 2:**
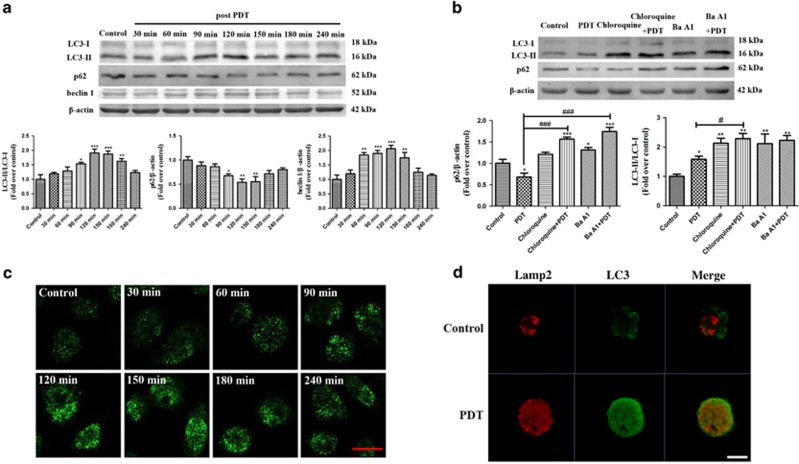
UCNPs-Ce6-mediated PDT is effective in inducing THP-1 macrophage-derived foam cell autophagy. (**a**) The expression levels of the autophagy-related proteins LC3, p62 and beclin 1 from 30 min to 6 h after PDT. (**b**) The effects of chloroquine and Ba A1 on the expression levels of the autophagy-related proteins LC3 and p62. (**c**) The relative fluorescence of LC3 from 30 min to 6 h after PDT was detected by LSCM (scale bar, 20 *μ*m). (**d**) The effect of PDT on LC3 and Lamp2 co-localization at 2 h after PDT, detected by LSCM (scale bar, 5 *μ*m). (*n*=3; *P<0.05, **P<0.01, ***P<0.001 *versus* control group. ^#^*P*<0.05, ^###^*P*<0.001 *versus* PDT group)

**Figure 3:**
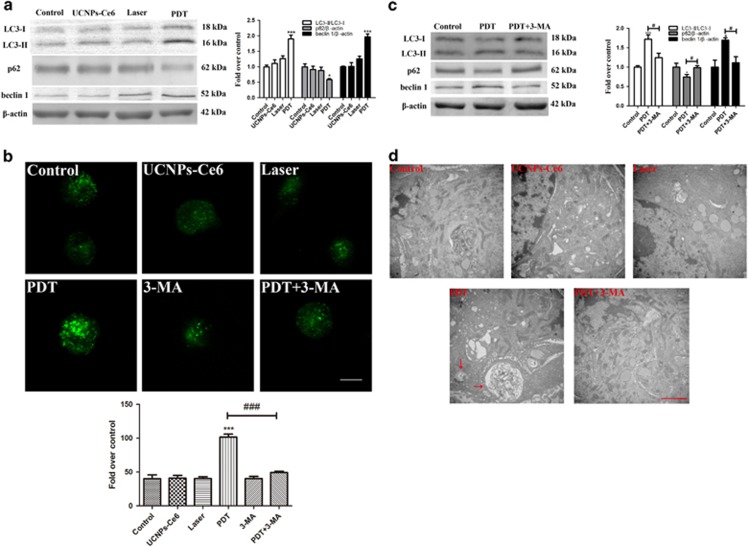
Autophagy induced by PDT is dependent on the combined effects of the laser and UCNPs-Ce6. (**a**) The expression levels of the autophagy-related proteins LC3, p62 and beclin 1 2 h after the various treatments. (**b**) The observation of AVOs induced by various treatments using MDC staining (scale bar, 5 *μ*m). (**c**) The effects of 3-MA on the expression levels of the autophagy-related proteins LC3, p62 and beclin 1 2 h after PDT. (**d**) Morphological alterations of THP-1 macrophage-derived foam cells following various treatments detected via representative transmission electron microscopy (red arrow: autophagic vacuoles with cytoplasmic contents, scale bar, 2 *μ*m); (*n*=3; *P<0.05, ***P<0.001 *versus* control group, ^###^*P*<0.001 versus PDT group)

Autophagy activation promotes the cholesterol efflux of macrophage foam cells.^20^ Thus, we examined whether PDT promoted cholesterol efflux via autophagy and found that PDT notably upregulated LC3-II and beclin 1 expression and downregulated p62 expression; effects that peaked 2 h after PDT (Figure 2a). Additionally, the relative fluorescence level of LC3 was evaluated to monitor autophagosome formation. As shown in Figure 2c, LC3 was distributed evenly throughout the cell in the control group, whereas PDT resulted in more distinctive LC3 spots that also peaked 2 h after PDT. Moreover, Lamp2 staining colocalized with the LC3-positive staining 2 h after PDT, indicating the formation of autophagosomes (Figure 2d). Further analysis also indicated an intact autophagic flux following PDT, as shown by further accumulation of LC3-II and p62 after pre-treatment with chloroquine and bafilomycin A1 (Ba A1) (Figure 2b). Figure 3a showed that laser or UCNPs-Ce6 alone had no significant effect on the expression of autophagy-related proteins. Additionally, monodansylcadaverine (MDC) staining resulted in brighter green fluorescence of the MDC-positive cells in the PDT group than in the other groups. Additionally, MDC staining results showed that green fluorescence of the MDC-positive cells in the PDT group is brighter than in the other groups, which could be attenuated by 3-methyladenine (3-MA) (Figure 3b). Pre-treatment with 3-MA also reversed the changes in autophagy-related proteins induced by PDT (Figure 3c). Moreover, the autophagy ultrastructure was observed by transmission electron microscopy 2 h after PDT. As shown in Figure 3d, cells treated with PDT exhibited typical myelin figures and autophagic vacuoles with cytoplasmic contents. However, the autophagic alterations were less evident in the other groups. Therefore, the combined effects of laser irradiation and UCNPs-Ce6 were responsible for autophagy induction in THP-1 macrophage foam cells.

**Figure 4:**
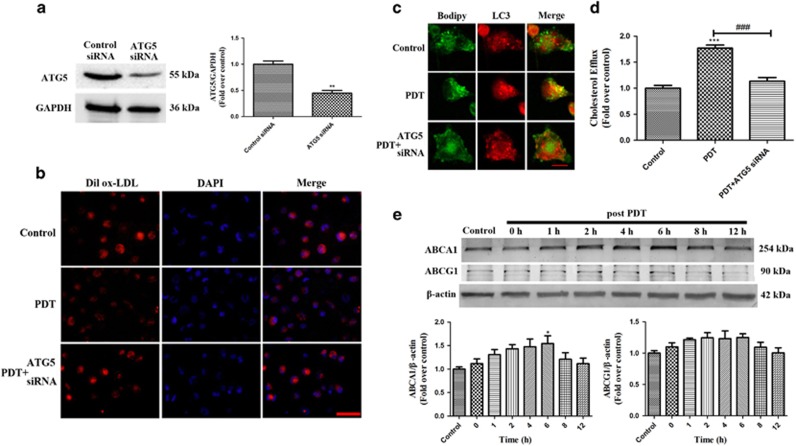
PDT-induced autophagy promotes the cholesterol efflux via ABCA1. (**a**) ATG5 expression was detected by western blotting. (**b**) The effect of ATG5 siRNA on the intracellular lipid burden was measured using Dil ox-LDL and DAPI staining (scale bar, 50 *μ*m). (**c**) The effect of ATG5 siRNA on the co-localization between LC3 and Bodipy 2 h following the different treatments, as detected by LSCM (scale bar, 10 *μ*m). (**d**) Quantitative detection of cholesterol efflux using a fluorometric assay at 6 h post-PDT. (**e**) The expression levels of the ATP-binding cassette transporters ABCA1 and ABCG1 at various time points after PDT (*n*=3; *P<0.05, **P<0.01 *versus* control group, ^###^*P*<0.001 *versus* PDT group)

To evaluate the relationship between PDT-induced autophagy and the cholesterol efflux, we blocked autophagy using an ATG5 siRNA (Figure 4a) and found that the co-localization between the Bodipy fluorescence and punctuated LC3-positive fluorescence was diminished (Figure 4c). Pre-treatment with the ATG5 siRNA significantly enhanced the intracellular lipid deposition and decreased the cholesterol efflux 6 h after PDT (Figures 4b and d), suggesting that UCNPs-Ce6-mediated PDT promoted cholesterol efflux via autophagy. ABCA1 and ABCG1 are major ATP-binding cassette transporters that have crucial roles in mediating cholesterol efflux.^21, 22^ We found that ABCA1 expression increased following PDT, whereas ABCG1 expression showed no obvious changes (Figure 4e), suggesting that the cholesterol efflux was mediated by ABCA1.

### UCNPs-Ce6-mediated PDT induced the autophagy of THP-1 macrophage-derived foam cells through the PI3K/Akt/mTOR pathway

**Figure 5:**
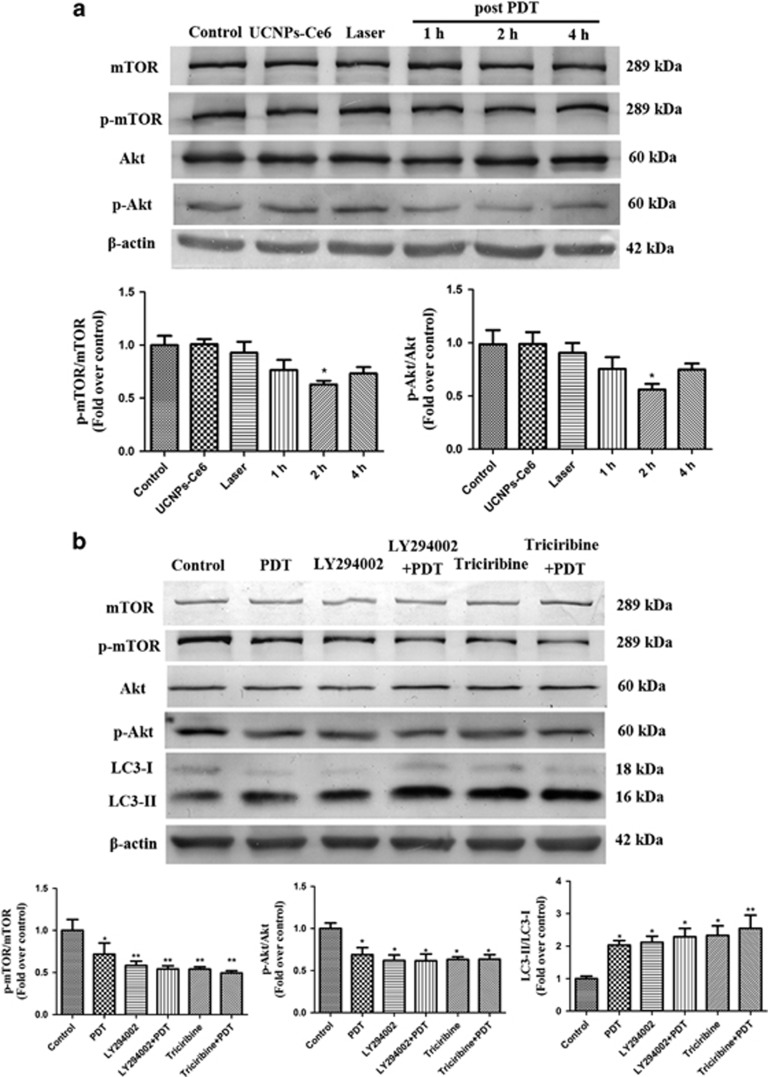
UCNPs-Ce6-mediated PDT induces autophagy through the PI3K/Akt/mTOR pathway. (**a**) The expression levels of PI3K/Akt/mTOR pathway-related proteins at various time points after PDT. (**b**) The effects of LY294002 and triciribine on the expression levels of PI3K/Akt/mTOR pathway-related proteins and the autophagy-related protein LC3 2 h after PDT. (*n*=3; *P<0.05, **P<0.01 *versus* control group)

The PI3K/Akt/mTOR pathway is closely associated with autophagy regulation.^23, 24^ Akt and mTOR phosphorylation levels were decreased significantly 2 h after PDT (Figure 5a). Additionally, pre-treatment with the PI3K inhibitor LY294002 or Akt inhibitor triciribine did not affect the expression of p-Akt, p-mTOR and LC3-II (Figure 5b). whereas pre-treatment with the PI3K activator IGF-1 reversed the enhancement of LC3-II induced by PDT (Supplementary Figure 3). Accordingly, UCNPs-Ce6-mediated PDT-induced autophagy though the PI3K/Akt/mTOR pathway.

### Activation of autophagy through the PI3K/Akt/mTOR pathway was regulated by ROS

**Figure 6:**
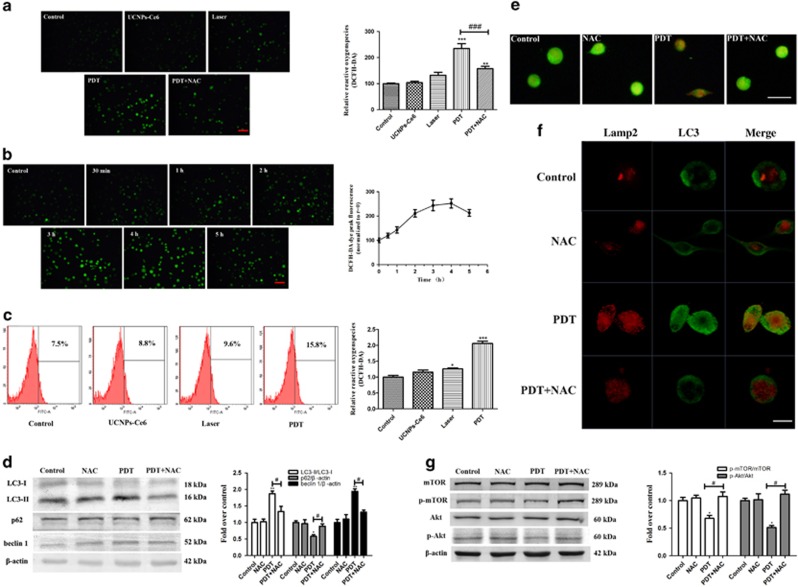
PDT-induced ROS has an important role in regulating autophagy via the PI3K/Akt/mTOR pathway. (**a**) Intracellular ROS generation of THP-1 macrophage foam cells was measured by DCFH-DA staining (scale bar, 50 *μ*m). (**b**) Alterations of intracellular ROS from 30 min to 5 h post-PDT. (**c**) Intracellular ROS generation measured by flow cytometry. (**d**) The effects of the ROS scavenger NAC on the expression levels of the autophagy-related proteins beclin 1, p62 and LC3 2 h after PDT. (**e**) The effect of NAC on AVO formation using acridine orange staining (scale bar, 50 *μ*m). (**f**) The effects of NAC on the co-localization of LC3 and Lamp2 2 h after the different treatments, as detected by LSCM (scale bar, 10 *μ*m). (**g**) The effects of NAC on the expression levels of PI3K/Akt/mTOR pathway-related proteins 2 h post-PDT (*n*=3; *P<0.05, **P<0.01 *versus* control group. ^#^*P*<0.05 *versus* PDT group)

ROS production is considered the main mechanism underlying PDT.^25, 26^ Thus, we measured ROS production using the intracellular ROS probe 2′,7′-dichlorofluorescein (DCF). As shown in Figure 6a, the green fluorescence increased slightly in the laser alone group, whereas significant increases in green fluorescence were observed in the PDT group. The increase in ROS production could be blocked by pre-treatment with NAC. Intracellular ROS production determined by flow cytometry also showed that ROS production increased significantly 2 h after PDT (Figure 6c). Next, we investigated the alteration in ROS within 5 h following PDT and found that ROS production increased in a time-dependent manner and peaked 4 h after PDT (Figure 6b).

Additionally, we investigated the effect of ROS on PDT-induced autophagy. Figure 6d shows that the changes in autophagy-related proteins (LC3, p62 and beclin 1) were reversed by pre-treatment with the ROS scavenger NAC. To further evaluate the interaction between autophagy and ROS, acridine orange staining was used to observe acidic vesicular organelles (AVOs). The cells treated with PDT exhibited more obvious red fluorescence than the control groups; this fluorescence could be attenuated with NAC pre-treatment (Figure 6e). Simultaneously, NAC diminished the PDT-induced co-localization between the punctate LC3-positive green fluorescence and the Lamp2 red fluorescence (Figure 6f). NAC also reversed the p-mTOR and p-Akt suppression induced by PDT (Figure 6g), suggesting that autophagy activation through the PI3K/Akt/mTOR pathway was regulated by ROS. To identify roles of different degree of ROS had, we detected the expression levels of LC3 and cleaved Caspase 9 (apoptosis-related protein) under different laser intensity (Supplementary Figure 4), data showed cell apoptosis was observed from 1.6 W/cm^2^ of laser, suggesting that exceed ROS could induce apoptosis in THP-1 macrophage-derived foam cells.

### PDT promoted ROS generation in peritoneal macrophage-derived foam cells that subsequently induced autophagy via suppression of the PI3K/AKT/mTOR pathway and promoted cholesterol efflux

**Figure 7:**
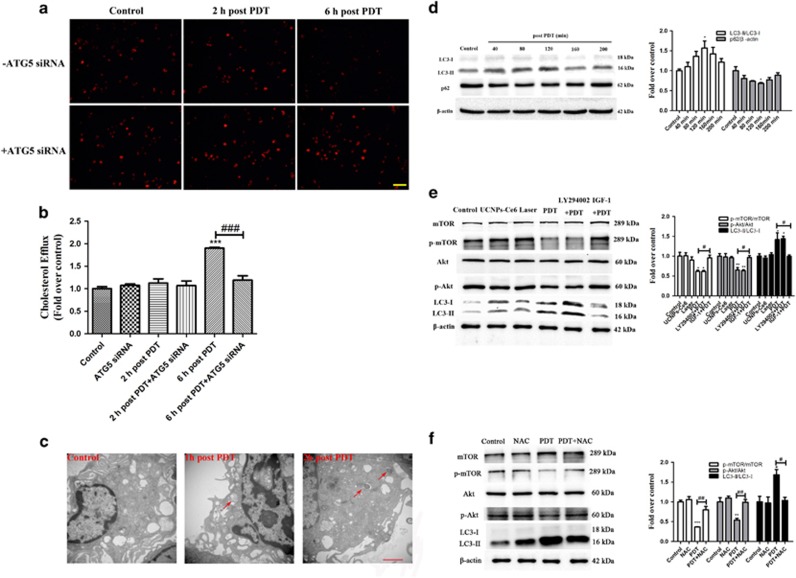
Photodynamic effects on peritoneal macrophage-derived foam cells. (**a**) The effect of PDT on the intracellular lipid burden measured by Dil ox-LDL staining (scale bar, 50 *μ*m). (**b**) Quantitative detection of the cholesterol efflux following PDT using a fluorometric assay. (**c**) Morphological alterations following PDT detected via representative transmission electron microscopy (red arrow: autophagic vacuoles with cytoplasmic contents, scale bar, 2 *μ*m). (**d**) The expression levels of the autophagy-related proteins p62 and LC3 from 40 min to 200 min post-PDT. (**e**) The effects of LY294002 and IGF-1 on the expression levels of PI3K/Akt/mTOR pathway-related proteins and the autophagy-related protein LC3 2 h post-PDT. (**f**) The effects of NAC on the expression levels of PI3K/Akt/mTOR pathway-related proteins 2 h post-PDT (*n*=3; *P<0.05, **P<0.01, ***P<0.001 *versus* control group, ^#^ *P*<0.05, ^##^*P*<0.01, ^###^*P*<0.001 *versus* PDT group)

We examined the photodynamic effect on peritoneal macrophage foam cells. Dil-labeled ox-LDL staining indicated that lipid storage decreased significantly starting 6 h after PDT (Figure 7a), whereas ATG5 siRNA abolished this effect. Cholesterol efflux was also quantitatively analysed using a fluorometric assay (Figure 7b). Collectively, these results indicate that PDT promotes the cholesterol efflux of peritoneal macrophage foam cells. Obvious morphological changes were observed in the PDT-treated foam cells, including increases in the numbers of autophagosomes and autolysosomes (Figure 7c). Additionally, autophagy was markedly increased following PDT based on the increase in the LC3-II/LC3-I ratio and the decrease in the p62 level (Figure 7d). The p-AKT and p-mTOR expression levels were significantly decreased post-PDT but could be reversed by IGF-1 (Figure 7e), suggesting that PDT promoted autophagy via suppression of the PI3K/AKT/mTOR pathway. Furthermore, the ROS scavenger NAC attenuated the increases in p-AKT p-mTOR and LC3-II (Figure 7f), indicating that autophagy activation through the PI3K/Akt/mTOR pathway was regulated by ROS.

## Discussion

Macrophage-derived foam cells are a major component of atherosclerotic plaques. This cell type has crucial roles in the formation of the necrotic core and the establishment of chronic inflammation, resulting in the progression and instability of atherosclerotic plaques.^27^ Thus, attenuating lipid deposition in macrophage foam cells is vitally important. PDT, which combines photosensitizers, light, and molecular oxygen, has been used for the treatment of various diseases. Our study has confirmed that 5-aminolevulinic-acid-mediated PDT postponed the progression of atherosclerosis by reducing the plaque content.^28^ Therefore, we utilized UCNPs-Ce6 as a novel composite material for PDT and further explored its effects on both THP-1 and peritoneal macrophage-derived foam cells *in vitro*.

First, we examined the intracellular lipid deposition and measured the cholesterol efflux induced by PDT. In this study, the intracellular lipid deposition decreased dramatically after PDT. In contrast, cholesterol efflux was less evident in the control groups (the control, laser and UCNPs-Ce6 alone groups). Based on this finding, we quantitatively estimated the cholesterol efflux using a fluorometric assay and observed that cholesterol efflux was increased significantly 6 h after PDT, which implied that UCNPs-Ce6-mediated PDT promoted cholesterol efflux of THP-1 macrophage foam cells.

Next, we investigated the potential mechanisms underlying the PDT-induced cholesterol efflux. Autophagy has emerged as an alternative lipid metabolic pathway through the lysosomal degradative pathway, making this process a potential therapeutic target for diseases related to lipid metabolism disorders.^18, 19^ Increasing evidence has indicated that various cell types initiate autophagic responses after PDT.^29, 30^ LC3-II is a reliable autophagosomal marker that leads to the elongation of the phagophore membrane. Beclin 1 also has a critical role in autophagosome formation by interacting with class III-type PI3K, and the p62 receptor protein is selectively degraded via autophagy.^31, 32^ Thus, detection of LC3-II, p62 and beclin 1 is used to monitor autophagosome formation and autophagic flux. This study confirmed that autophagy induction was increased significantly and peaked 2 h after PDT, as demonstrated by the increase in LC3-II detected in both the western blotting and immunofluorescence assays. Additionally, an increase in beclin 1 and a decrease in p62 expression were observed following PDT. Furthermore, to monitor the autophagic flux, the LC3 and p62 levels were measured in the presence of Ba A1 and chloroquine, which inhibited organelle acidification and subsequently inhibited autophagosome and lysosome fusion.^33^ Pre-treatment with Ba A1 and chloroquine in the PDT group resulted in further LC3-II and p62 accumulation compared with the cells treated with Ba A1 and chloroquine alone. Moreover, a direct association between LC3 and Lamp2 was observed by immunofluorescence after PDT. However, the laser and UCNPs-Ce6 alone groups had no obvious impact on the expression of autophagy-related proteins, AVO staining or autophagic ultrastructure formation compared with the control group. Therefore, we confirmed that UCNPs-Ce6-mediated PDT was responsible for autophagy induction in THP-1 macrophage foam cells. To investigate whether autophagy was involved in the cholesterol efflux process, we utilized an ATG5 siRNA to block autophagy and Bodipy to fluorescently stain lipids. Pre-treatment with the ATG5 siRNA not only significantly diminished the co-localization between LC3-positive staining and Bodipy but also attenuated the cholesterol efflux induced by PDT. Collectively, these results indicate that the PDT-induced cholesterol efflux is related to autophagy induction.

The fusion of autophagosomes with lysosomes during the autophagic process is a vital step that leads to the degradation of their cargo.^34^ Cholesterol esters are believed to be hydrolyzed into free cholesterol by lysosomal acid lipase, and the free cholesterol is then delivered to the periphery via ATP-binding cassette transporters.^35, 36^ ABCA1 and ABCG1 are major transporters that have crucial roles in mediating cholesterol efflux.^21, 22^ Thus, we examined whether ABCA1 and ABCG1 were involved in the PDT-induced cholesterol efflux. PDT increased ABCA1 expression but did not significantly alter ABCG1 expression, which implied that cholesterol efflux might be mediated by ABCA1.

Our study contributed toward the elucidation of the mechanism of UCNPs-Ce6-mediated PDT via autophagy activation. The PI3K/Akt/mTOR signaling pathway is a classic autophagy pathway.^23, 24^ PI3K and Akt suppression can inhibit mTOR phosphorylation at the Ser^2448^ site, thereby enhancing the expression of autophagy-related proteins and inducing autophagy.^37^ To determine whether PDT-induced autophagy occurred through the PI3K/Akt/mTOR pathway, we monitored the Akt and mTOR phosphorylation at different time points after PDT and found that mTOR and Akt phosphorylation significantly decreased after PDT. Additionally, pre-treatment with the PI3K inhibitor LY294002 or the Akt inhibitor triciribine did not affect the expression of p-Akt, p-mTOR and LC3-II. Conversely, pre-treatment with the PI3K activator IGF-1 significantly reversed the enhancement of LC3-II induced by PDT. These results suggested that UCNPs-Ce6-mediated PDT-induced autophagy through the PI3K/Akt/mTOR pathway.

**Figure 8:**
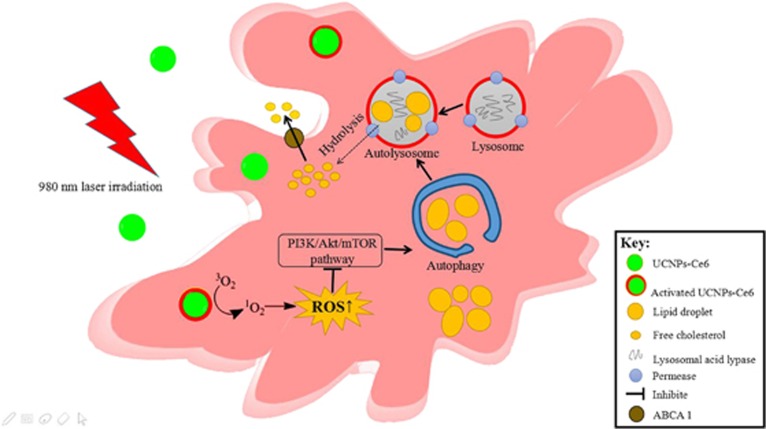
Schematic diagram of UCNPs-Ce6-mediated PDT and the mechanism of autophagy.Notes: UCNPs-Ce6-mediated PDT exhibits an autophagic response through the PI3K/Akt/mTOR signaling pathway that is regulated by ROS, which causes the ABCA1-dependent cholesterol efflux

As major products of PDT, ROS are metabolites of oxygen that serve as essential mediators in many biological processes and regulate oxidation-reduction reactions, senescence and cell demise.^38^ Intracellular ROS production increased following PDT and could be blocked by the ROS scavenger NAC. Although ROS have been reported to initiate autophagy, the interplay between ROS and autophagy remains unknown.^39^ Thus, we investigated whether autophagy induction was related to ROS production. Here, we observed that PDT-induced autophagy could be significantly inhibited by the ROS inhibitor NAC. Pre-treatment with NAC reversed the effects of PDT on autophagy-related proteins. Simultaneously, NAC reduced AVO formation and diminished the co-localization between LC3 and Lamp2 induced by PDT, indicating that ROS had an essential role in autophagy induction. Furthermore, the p-Akt and p-mTOR suppression induced by PDT was reversed by pre-treatment with NAC, which implied that the PI3K/Akt/mTOR pathway was regulated by ROS production. We also demonstrated that PDT promoted ROS generation in peritoneal macrophage foam cells, which subsequently induced autophagy via suppression of the PI3K/AKT/mTOR pathway and promoted cholesterol efflux. Taken together, ROS produced by PDT could activate foam cell autophagy via inhibiting PI3K pathways, then the lipids were degraded into free cholesterol by autophagy and released from the cell via ABCA1 (Figure 8).

In general, our findings provide a novel mechanism in which a PDT-induced increase in autophagy benefits the cholesterol efflux. Moreover, the ROS/PI3K/Akt/mTOR signaling pathway was determined to be important in PDT-induced autophagy. Therefore, UCNPs-Ce6-mediated PDT may represent a potential strategy against atherosclerotic plaque progression.

## Materials and methods

### The 980-nm PDT device

The 980-nm PDT device used in this study was developed by the Harbin Institute of Technology (Harbin, China). The laser generator emits a single 980-nm wavelength irradiation with a 2-mm diameter. For PDT treatment, the cells were placed 2 cm below the laser generator with a laser irradiation density of 1.0 W/cm^2^. During this procedure, the temperature of the solution inside the cultures increased by less than 2 °C.

### Cell culture and foam cell formation

The human THP-1 monocytic leukaemia cell line was purchased from the American Type Culture Collection (Manassas, VA, USA). The cells were cultured in RPMI 1640 medium (HyClone, Logan, UT, USA) containing 10% fetal bovine serum (FBS, HyClone), 20 *μ*g/ml of penicillin, and 20 *μ*g/ml of streptomycin (Sigma-Aldrich, St. Louis, MO, USA). The cells were maintained at 37 °C in a humidified incubator with 5% CO_2_. For the experiments, the cells were seeded in 35-mm Petri dishes or 96-well plates at a density of 1.0 × 10^5^ cells per milliliter and differentiated into macrophages with the addition of 100 ng/ml of phorbol-12-myristate-13-acetate (PMA, La Jolla, CA, USA) for 72 h. Macrophages were transformed into foam cells by adding 50 *μ*g/ml of ox-LDL (Union-Biology, Beijing, China) to serum-free RPMI 1640 medium containing 0.3% bovine serum albumin (BSA, Hyclone) for 48 h.

Peritoneal macrophages were collected from C57 mice 48 h following injection with 5 ml of 3% thioglycollate broth medium into the peritoneum. The cells were then transformed into foam cells by adding 50 *μ*g/ml of ox-LDL in serum-free RPMI 1640 medium containing 0.3% BSA for 48 h.

### PDT treatment protocols

UCNPs-Ce6 was designed by the Harbin Institute of Technology.^40-42^Approximately 660 *μ*g/ml of stock solution in dimethyl sulfoxide was stored as aliquots at 4 °C in the dark.

Ox-LDL-derived macrophage foam cells were collected, differentiated and randomly divided into four groups as follows: control, UCNPs-Ce6 alone, laser alone and PDT. For both the UCNPs-Ce6 and PDT groups, the cells were incubated with 8 *μ*g/ml of UCNPs-Ce6 for a drug loading time of 4 h in FBS-loaded RPMI 1640 medium. For the control and laser alone groups, an equivalent volume of medium was added to replace the UCNPs-Ce6. The cells in both the laser alone and PDT groups were exposed to 980-nm laser irradiation with an intensity of 1.0 W/cm^2^ for 60 s. After treatment, the cells were washed once with phosphate-buffered saline (PBS), cultured in fresh medium for an additional 2 h and then prepared for the different analyses.

For the inhibitory studies, 1 mM of the ROS inhibitor NAC (Sigma-Aldrich), 10 mM of the autophagy inhibitor 3-MA (Sigma-Aldrich), 50 *μ*M of the autophagy inhibitor chloroquine (Sigma-Aldrich), 100 nM of the autophagy inhibitor Ba A1 (Sigma-Aldrich) and 10 ng/ml of the PI3K activator insulin-like growth factor 1 (IGF-1; Sigma-Aldrich) were incubated together with UCNPs-Ce6 for 4 h. The cells were treated with 10 mM of the PI3K inhibitor LY294002 (Sigma-Aldrich) and 20 mM of the Akt inhibitor triciribine (Sigma-Aldrich) for 24 h before incubation with UCNPs-Ce6.

### Cell viability assay

The cell survival rate was assessed using the CCK-8 assay (Beyotime Biotechnology, Beijing, China). THP-1 monocytes treated with 100 ng/ml of PMA were seeded into 96-well plates and incubated for 72 h to differentiate into THP-1 macrophages at 37 °C in the dark. Then, both THP-1 and peritoneal macrophages were transformed into foam cells by adding 50 *μ*g/ml of ox-LDL for 48 h. At the indicated time points after the different treatments, the medium was removed carefully, and 100 *μ*l of medium containing CCK-8 (RPMI 1640 medium and CCK-8 volume ratio of 9:1) was added to each well. After incubation for 1 h at 37 °C in the dark, the absorption of each well at 450 nm was measured with a microplate reader (Varian Australia Pty, Australia). The data represent the averages of six wells per group. Each experiment was repeated at least three times individually.

### Detection of intracellular ROS

Intracellular ROS were analysed by measuring the fluorescence intensity of DCF and via flow cytometry analysis. At the indicated time points following PDT, the cells were washed twice with PBS and then incubated with 20 *μ*M 2′-7′-dichloroflorescein diacetate (DCFH-DA) diluted in serum-free medium for 30 min at 37 °C in the dark. Then, the cells were washed twice with PBS. Immediately after washing, the cells were measured via fluorescence spectrophotometry (Varian Australia Pty) at excitation and emission wavelengths of 488 nm and 525 nm, respectively.

For the flow cytometry assay, the cells were incubated with the probe for 30 min in serum-free medium at 37 °C in the dark. Then, the samples were analysed using a FACSCalibur flow cytometer (BD Biosciences).

### Transmission electron microscopy examination

At 2 h post-PDT, the cells were harvested by centrifugation and fixed with 2.5% glutaraldehyde in 100 mM cacodylate overnight at 4 °C. The cells were then washed twice with PBS and placed in 1% osmium tetroxide (OsO_4_) in 100 mM cacodylate buffer for 2 h. After washing 3 times with distilled water, the cells were dehydrated in a graded series of ethanol solutions and isoamyl acetate for 15 min. Then, the cells were microsectioned into ultra-thin sections and stained with uranyl acetate and lead citrate for observation using a transmission electron microscope (JEM-1220; Japan).

### Immunofluorescence staining

The cells were seeded into 20-mm glass-bottom cell culture dishes. To detect autophagy activation, we utilized Bodipy to fluorescently stain lipids and measured co-localization between LC3 and the lysosomal marker Lamp2. To detect cholesterol efflux, we also measured co-localization between LC3 and Bodipy. At the indicated time points after PDT, the cells were washed twice with PBS, fixed with 4% paraformaldehyde for 10 min and permeabilized using 1% Triton X-100 for 5 min at room temperature. After washing with PBS, the cells were blocked with 3% BSA to prevent non-specific antibody binding. The cells were incubated with diluted antibodies (1:400) against Lamp2 and LC3 overnight at 4 °C. The cells were washed twice with PBS and incubated with diluted (1:200) tetramethylrhodamine isothiocyanate (TRITC)- or fluorescein isothiocyanate (FITC)-labeled secondary rabbit antibodies, Dylight 405-labeled secondary rabbit antibodies or 10 *μ*g/ml of Bodipy in 1% BSA at room temperature for 1 h in the dark. After washing twice with PBS, the cells were observed under a laser scanning confocal microscope (LSCM; LSCM 510 Meta; Zeiss, Gottingen, Germany) with the appropriate lasers.

### MDC staining

MDC (Sigma-Aldrich) was used as an autolysosome marker to observe autophagy formation. At 2 h after the different treatments, the cells were incubated with 50 *μ*mol/ml of MDC diluted with RPMI 1640 medium for 30 min at 37 °C in the dark. After washing twice with PBS, the cells were visualized and photographed under a fluorescence microscope (IX-71; Olympus, Tokyo, Japan).

### Acridine orange staining

Acridine orange (Sigma-Aldrich) staining was used to observe acidic vesicle organelles (AVOs), which represent autophagy formation. At 2 h after the different treatments, the cells were incubated with 0.01% acridine orange diluted with distilled water for 5 min at 37 °C in the dark. After washing twice with PBS, the cells were visualized and photographed under a fluorescence microscope (IX-71; Olympus).

### Cholesterol efflux assessment

Oil red O and Dil ox-LDL staining were used to detect lipid accumulation in the cells. For the oil red O staining, at the indicated post-PDT time points, the cells were stained with freshly diluted 0.5% oil red O solution for 10 min at 37 °C. After washing twice with PBS, the cells were visualized and photographed under a fluorescence microscope (IX-71; Olympus). For the Dil ox-LDL staining, THP-1 macrophages were incubated with 30 *μ*g/ml of Dil ox-LDL diluted with RPMI 1640 medium for 6 h at 37 °C in the dark. After washing twice with PBS, the cells were incubated with 5 *μ*g/ml of 4',6-diamidino-2-phenylindole (DAPI) for 15 min in the dark. Then, the cells were washed with PBS, visualized and photographed under a fluorescence microscope.

The cholesterol efflux in the cells was quantified using the Cholesterol Efflux Fluorometric Assay Kit (Biovision, San Francisco, USA) according to the manufacturer’s protocol. The cholesterol efflux of the treatments was calculated by dividing the fluorescence intensity of the medium by the total fluorescence intensity of the cell lysate and the medium.

### Western blotting analysis

After the designated treatments, RIPA lysis buffer was used to extract total proteins from the cells. The concentrations of the collected proteins were assessed using a bicinchoninic acid (BCA) kit (Beyotime Biotechnology, Beijing, China). After electrophoresis on a polyacrylamide gel, protein samples (50 *μ*g) were transferred onto a polyvinylidene difluoride membrane (PVDF). After blocking with blocking buffer containing 5% low-fat milk diluted with Tris-buffered saline-Tween 20 (TBST), the membranes were incubated with primary antibodies at 4 °C overnight. After washing with TBST, the membranes were incubated with alkaline phosphatase (AP)-IgG or horseradish peroxidase (HRP)-conjugated secondary antibodies for 2 h at room temperature. After washing with TBST again, the immune complexes were detected using an enhanced chemiluminescence reagent. The protein bands were quantified using a Bio-Rad ChemiDoc EQ densitometer and Bio-Rad Quantity One software (Bio-Rad Laboratories, Hercules, CA, USA).

Antibodies against beclin 1, p62, LC3, mTOR, p-mTOR, Akt, p-Akt, ATG5, ABCA1 and ABCG1 (1:1000) were purchased from Cell Signaling Technology (Boston, USA). Antibodies against *β*-actin and GAPDH (1:1000) were purchased from Proteintech Group (Wuhan, China). The AP-IgG secondary mouse and rabbit antibodies (1:1000) and HRP-conjugated secondary mouse and rabbit antibodies (1:8000) were purchased from ZhongShan Company (Beijing, China).

### Small interfering RNA

The ATG5 and mTOR genes were silenced by targeting siRNA oligonucleotides to the cDNA sequence of the human ATG5 gene according to the manufacturer’s instructions. An irrelevant 21-nucleotide siRNA (GenePharma, Shanghai, China) was used as the negative control. The target sequences were as follows: sense: GCAGUGGCUGAGUGAACAUTT and antisense: AUGUUCACUCAGCCACUGCTT.

### Statistical analysis

All experiments were replicated at least three times independently. Differences between groups were analysed using one-way analysis of variance (ANOVA) and the independent sample *T* test. The results are presented as the mean±standard deviation (S.D.). Values that reached a level of significance of *P*<0.05 were considered significant.
