# Supplementary material for: Upconversion nanoparticle-mediated photodynamic therapy induces autophagy and cholesterol efflux of macrophage-derived foam cells via ROS generation
Source: Cell Death Dis. 2017 Jun 8;8(6):e2864–. doi: 10.1038/cddis.2017.242 (PMC5520901; doi:10.1038/cddis.2017.242)
Supplement: Supplementary Material [file cddis2017242x1.doc]

**Figure S1** The survival rate of THP-1 macrophage foam cells, as detected using the CCK-8 assay. (a) The survival rates of THP-1 macrophage foam cells incubated with different UCNPs-Ce6 concentrations for 4 hours. (b) The survival rates of THP-1 macrophage foam cells following incubation with 8 μg/mL UCNPs-Ce6 for various times. (c) The survival rates of THP-1 macrophage foam cells after 60 seconds of laser irradiation with different laser densities. (d) The survival rates of THP-1 macrophage foam cells following various laser irradiation times with a laser density of 1.0 W/cm^2^. (e) The survival rates of THP-1 macrophage foam cells following exposure to a 1.0 W/cm^2^ laser density and 60 seconds of laser irradiation with 8 µg/mL of UCNPs-Ce6 (n=3,^*^ *P*<0.05, ^**^ *P*<0.01, ^***^ *P*<0.001 vs control group).

**Figure S2** The survival rate of peritoneal macrophage foam cells, as detected using the CCK-8 assay. (a) The survival rates of peritoneal macrophage foam cells following incubation with 8 μg/mL of UCNPs-Ce6 for various times. (b) The survival rates of peritoneal macrophage foam cells following exposure to 1.0 W/cm^2^ laser density and 60 seconds of laser irradiation. (c) The survival rates of peritoneal macrophage foam cells following exposure to 1.0 W/cm^2^ laser density and 60 seconds of laser irradiation with 8 µg/mL of UCNPs-Ce6.

**Figure S3** IGF-1 reversed the autophagy induced by PDT. The effects of IGF-1 on the expression levels of PI3K/Akt/mTOR pathway-related proteins and the autophagy-related protein LC3 at 2 hours post-PDT (n=3,^*^ *P*<0.05, ^**^ *P*<0.01 vs control group, ^#^ *P*<0.05, ^##^*P*<0.01, ^###^*P*<0.001 vs PDT group).

**Figure S4** The expression of LC3 and cleaved Caspase 9 under different laser densities. The expression levels of LC3 and cleaved Caspase 9 of THP-1 macrophage foam cells following incubation with 8 μg/mL of UCNPs-Ce6 for various laser densities. (n=3, ^***^ *P*<0.001 vs control group).
