# Supplementary figures and images for: Upconversion nanoparticle-mediated photodynamic therapy induces autophagy and cholesterol efflux of macrophage-derived foam cells via ROS generation
Source: Cell Death Dis. 2017 Jun 8;8(6):e2864–. doi: 10.1038/cddis.2017.242 (PMC5520901; doi:10.1038/cddis.2017.242)

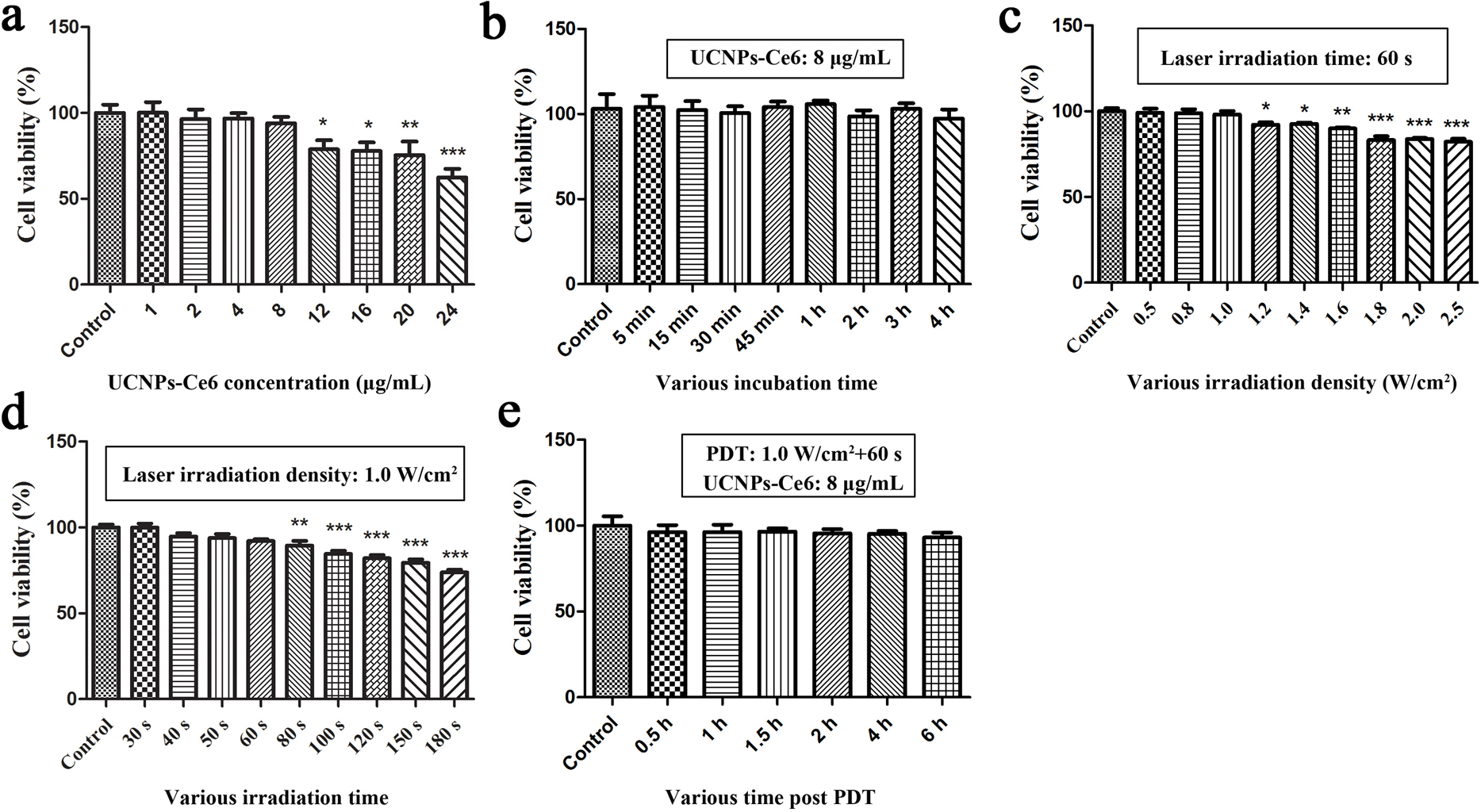

Supplement: Supplementary Figure 1 [file cddis2017242x2.tif]

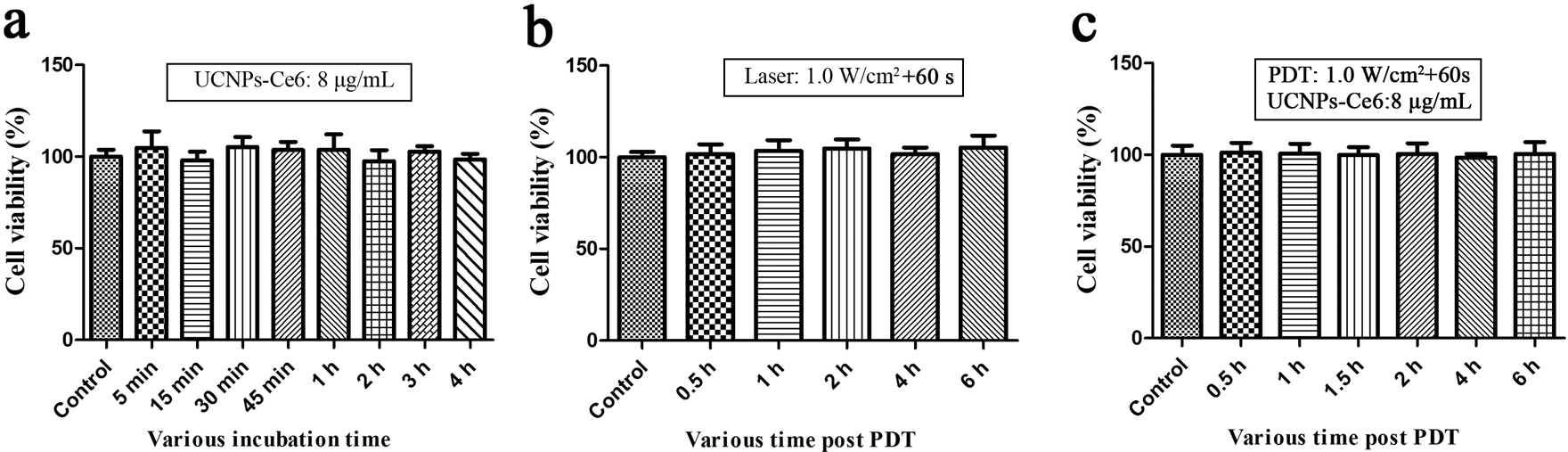

Supplement: Supplementary Figure 2 [file cddis2017242x3.tif]

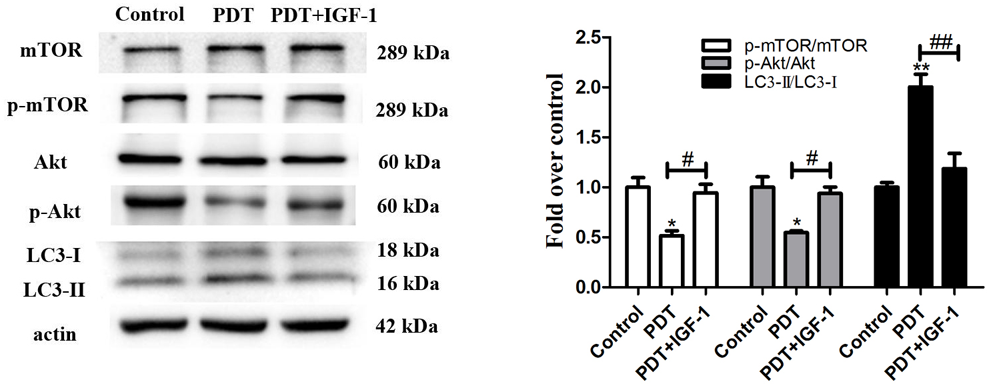

Supplement: Supplementary Figure 3 [file cddis2017242x4.tif]

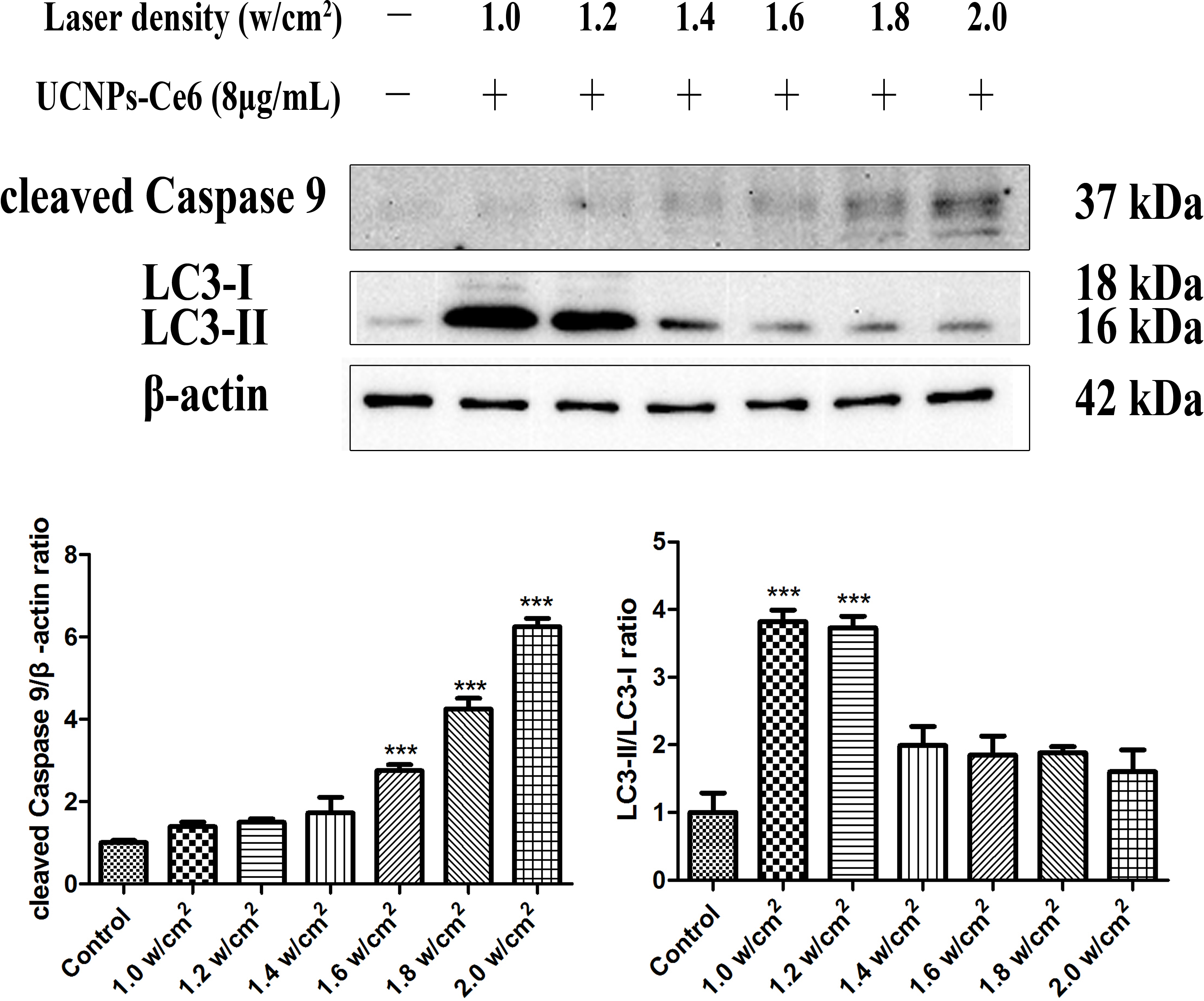

Supplement: Supplementary Figure 4 [file cddis2017242x5.tif]
